# Effects of drying strategies on sporulation and titer of microbial ecological agents with *Bacillus subtilis*

**DOI:** 10.3389/fnut.2022.1025248

**Published:** 2022-09-27

**Authors:** Chonglei Li, Kai Zhao, Litong Ma, Ji Zhao, Zhi-Min Zhao

**Affiliations:** ^1^Inner Mongolia Key Laboratory of Environmental Pollution Control and Wastes Reuse, School of Ecology and Environment, Inner Mongolia University, Hohhot, China; ^2^Key Laboratory of Ecology and Resource Use of the Mongolian Plateau (Ministry of Education), Inner Mongolia University, Hohhot, China; ^3^Inner Mongolia Engineering Research Center of Comprehensive Utilization of Bio-coal Chemical Industry, School of Chemistry and Chemical Engineering, Inner Mongolia University of Science and Technology, Baotou, China

**Keywords:** microbial ecological agents, *Bacillus subtilis*, sporulation, hot air drying, vacuum freeze drying, low-field nuclear magnetic resonance

## Abstract

Drying operation is beneficial to the preservation and transportation of microbial ecological agents. In this study, drying kinetics and water distribution variations in solid biomass medium during hot air drying (HAD) and vacuum freeze drying (VFD) were systematically investigated. Meanwhile, the effects of different drying strategies on the sporulation of *Bacillus subtilis* and the titer of microbial ecological agents were compared. The results showed that both HAD and VFD induced rapid water removal from the solid biomass medium. VFD retained bound water and maintained the porous structure of the solid medium. Both HAD and VFD induced sporulation. The expression level of sporulation-regulatory genes *spo0A, sigF*, and *sigE* followed the order 80°C-HAD > 60°C-HAD > VFD. The spore number in the medium after 80°C-HAD drying for 6 h was 0.72 × 10^10^/g dry medium, which was 9.1 and 12.5% larger than that of the medium with 60°C-HAD and VFD, respectively. Therefore, 80°C-HAD is an effective drying strategy for promoting sporulation, which improves the titer of microbial ecological agents with *B. subtilis*.

## Introduction

The indiscriminate use of antibiotics leads to the occurrence of antibiotic resistance genes (ARGs) and antibiotic-resistant bacteria (ARB) from environments (1, 2). *Bacillus subtilis* microbial ecological agents are environmentally friendly alternatives to antibiotics, which can be utilized as feed additives for preventing disease and promoting animal growth (3–5). The preparation process of microbial ecological agents consists of strain breeding, fermentation, and drying processes, among which the drying process aims to reduce water content (WC) in the solid substrate and enhance the stability of probiotics products for preservation and transportation (6, 7). Hot air drying (HAD) is widely used for the production of dehydrated food and solid medium. Vacuum freeze-drying (VFD) method sublimates water at subfreezing temperatures. It is the preferred drying method for biomaterials because most biomaterials are vulnerable to heat (8). However, there was rare information about the effects of HAD and VFD on the sporulation and titer of *B. subtilis* microbial ecological agents.

Low-field nuclear magnetic resonance (LF-NMR) has been regarded as a powerful tool to analyze the water states and distribution in the solid medium due to its fast analysis speed, good sensitivity, and non-invasiveness (9, 10). Using the LF-NMR technique, Li et al. (11) identified three different proton fractions (*T*_2*b*_, *T*_21_, and *T*_22_) with various mobilities in the glutinous rice during solid-state fermentation (SSF). Recently, the LF-NMR technique has exhibited an excellent ability to explore the water dynamics during the drying process for solid substrates (12). Bacteria spore number is a key indicator to evaluate the quality of microbial ecological agents (13). By forming spores, bacteria cells can survive in severe environments (e.g., acid and mechanical force) and reawaken when favorable conditions return (14). Spores of *B. subtilis* are resistant to environmental stress, which includes heat in the drying process (15). The sporulation process begins with the activation of the master transcriptional gene *spo0A*, which leads to the sequential activation of the *sigF* gene in the forespore and the *sigE* gene in the parent cell (16). Therefore, the expression of *spo0A, sigF*, and *sigE* genes can indicate the sporulation process of *B. subtilis* with different drying strategies.

This study aimed to compare the effects of different drying strategies on the sporulation of *B. subtilis* and improved the titer of microbial ecological agents. The key metrics, such as water dynamics, molecular genetic expressions, viable cell, and spore number variations, were systematically evaluated and compared. These together could help to reveal the relationship between water distribution and sporulation during different drying processes, which could further benefit the production of microbial ecological agents of *B. subtilis*.

## Materials and methods

### Solid-state fermentation

*Bacillus subtilis* 10732 was purchased from the China Center of Industrial Culture Collection (CICC). The strain was stored in Luria-Bertani (LB) medium slant at 4°C. *B. subtilis* 10732 was precultivated in 100 ml of a liquid medium that contained 1% (w/w) yeast extract, 4% (w/w) glucose, 1% (w/w) peptone, 1% (w/w) CaCO_3_, and 0.05% (w/w) MgSO_4_ at 37°C and was shaken at 150 rpm for 24 h.

The solid medium consisted of 3.0 g of soybean meal, 30 g wheat bran, 0.75 g glucose, 1.5 g CaCO_3_, 30 ml of 1.25% (w/w) (NH_4_)_2_SO_4_, 0.05% (w/w) MnSO_4_, 2.5% (w/w) KH_2_PO_4_ inorganic salt solution, and 9.6 ml of deionized water. The medium was put into 250 ml Erlenmeyer flasks and sterilized at 121°C for 20 min. Afterward, the medium was cooled at room temperature and then inoculated by adding 12 ml of seed solution. The fermentation was conducted in an incubator at 37°C for 72 h.

### Drying experiment

After fermentation, the solid medium was subjected to different drying treatments. The solid biomass medium was dehydrated until reaching constant weight. The temperatures of HAD were set at 60 and 80°C. The process of VFD included two steps: freezing and drying. All water in the solid medium was frozen in the first freezing step at −80°C for 1 h. The drying step was set at −45°C and 10 Pa to remove water in a vacuum environment until the medium reached a constant weight (8, 17).

Water content and drying rate (DR) are two important indicators to reveal the drying performance (12, 18). During the drying process, the mass of the solid medium was weighed, and the WCs (dry basis, g·g^−1^) were calculated using Equation (1):


(1) $$\begin{array}{l} WC_{t}=\left(m_{t}-m_{e}\right)/m_{e} \end{array}$$

where *WC*_*t*_ is the WC (g·g^−1^) at the time *t, m*_*t*_ is the mass (g) of the solid medium at *t* h, and *m*_*e*_ is the final dry mass (g) of the solid medium. DR (g·g^−1^·h^−1^) was calculated according to Equation (2):


(2) $$\begin{array}{l} DR=\left(WC_{t}-WC_{t+\text{Δ}t}\right)/{\text{Δ}}_{t} \end{array}$$

where *WC*_*t*_ and *WC*_*t*+Δ*t*_ are the WC at *t* and *t* + Δ*t*, respectively. The samples were taken at intervals of 1 h for WC and DR analyses.

### LF-NMR measurements

Low-field nuclear magnetic resonance was applied to analyze water states and dynamics in a solid medium on a MesoMR23-060H-I Analyst Analyzing and Imaging System (Niumag Co., Ltd., Shanghai, China). In total, 2.60 g of solid medium samples were obtained from the central part of the substrates. The samples were placed into 25 mm NMR glass tubes first and then inserted into the NMR probe for detection. The *T*_2*i*_ relaxation times were measured using Carr-Purcell-Meiboom-Gill (CPMG) sequence with a τ-value of 100 μs. A total of 3,000 echoes were collected as 16 scan repetitions, and the repetition time between two successive scans was 2.5 s. The relaxation time measurements were performed at 32°C. Data analysis was performed with the NMR Analyzing System version 1.0 (Niumag Co., Ltd., Shanghai, China). Relaxation time *T*_2*i*_ and its corresponding water proportion *M*_2*i*_ were presented.

### Measurement of viable cell and spore number

Approximately 3 g of the solid medium was mixed with sterile water (1:20, w/v) in a 250 ml Erlenmeyer flask and shaken at 150 rpm for 30 min at 37°C. In total, 100 μl of the supernatant was carefully sampled and serially diluted (10^6^-fold). Afterward, the cell count was determined by the spread plate method on LB plates. After incubation at 37°C for 24 h, the viable cell number was counted and expressed as colony-forming units (CFUs). Similarly, 5.0 ml of the mixture after shaking at 150 rpm for 30 min at 37°C in the determination of viable cell number was sampled and cultured at 80°C for 15 min. Afterward, 100 μl of the mixture was serially diluted (10^6^-fold) and spread on LB plates. After incubation at 37°C for 24 h, the spore number was determined.

### Quantitative polymerase chain reaction (qPCR) analysis of *spo0A, sigF*, and *sigE* gene expressions

Approximately 1 g of the solid medium was sampled and mixed with sterile water (1:20, w/v) in a 50 ml sterile test tube and shaken at 150 rpm for 5 min. Afterward, the mixture was centrifuged at 8,000 rpm for 10 min, and the precipitate was separated and stored at −80°C. Total RNA of *B. subtilis* was extracted using Cetyltrimethylammonium Bromide (CTAB) method (19). Before reverse transcription, the purity of the total RNA was tested by measuring OD260 and OD280 with a Nano Photometer P-Class P360 Ultraviolet Spectrophotometer. In total, 10 μl of total RNA was used as a template and was reverse transcribed to cDNA using PrimeScript RT Regent Kit (Takara Company) with a final volume of 20 μl. Moreover, the mixture was incubated at 37°C for 15 min and then at 85°C for 5 min to inactivate the enzyme. The primers for qPCR were synthesized by Sangon Biotech (Shanghai, China). The qPCR procedure was 30 s at 95°C followed by 35 cycles of 30 s at 95°C, 45 s at the annealing temperature of 35°C, and 45 s at 72°C, with a melt curve increasing from 65 to 95°C at increments of 0.5°C. The sample dried for 0 h was selected as the calibration sample for the gene expressions of *spo0A, sigF*, and *sigE*. The relative quantification of gene transcription was analyzed using the 2^−Δ*ΔCt*^ method (20).

### Statistical analysis

Statistical analysis was performed by using SPSS version 21.0. Differences between the means were evaluated by the one-way ANOVA and the independent samples *t-*tests.

## Results and discussion

### Changes of WC and DR during different drying processes

**Figure 1:**
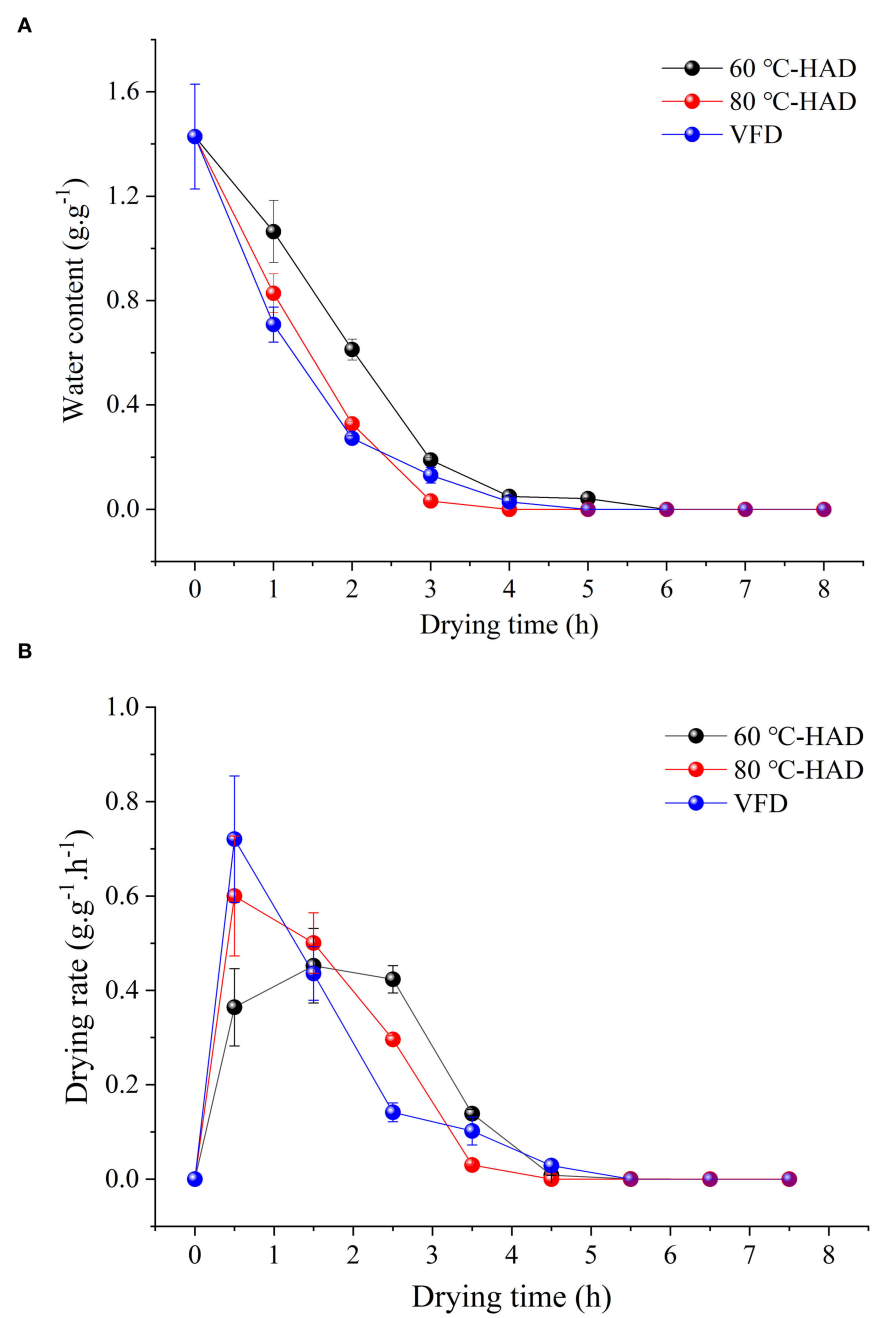
Changes in water content **(A)** and drying rate **(B)** during different drying processes.

Water content and DR are two important indicators to evaluate the drying process of a solid medium. Effects of different drying strategies on WC and DR were investigated (Figure 1). The WC of all media was decreased during the drying processes. The solid medium reached constant weight after 4 h drying with 80°C-HAD, while the time needed to reach constant weight with VFD and 60°C-HAD was 5 and 6 h, respectively. The higher the drying temperature, the faster the WC of the solid medium declined (12). Figure 1B shows that DR of 80°C-HAD and VFD increased from 0 to 1 h and then decreased gradually to the end of the drying process. The maximum DR values of 80°C-HAD and VFD were 0.60 and 0.72 g·g^−1^·h^−1^, respectively. The highest DR of 60°C-HAD was 0.45 g·g^−1^·h^−1^, which appeared during the period of 1–2 h. Sun et al. (18) reported that there were three stages during microwave freeze-drying for raspberry: the speed-up drying stage (I), the speed-down drying stage (II), and the low-speed drying stage (III), respectively. In line with the previous study, the drying process of solid biomass medium with HAD and VFD in this study could also be divided into three periods: (I) the speed-up drying period (0–1 h for 80°C-HAD and VFD and 0–2 h for 60°C-HAD); (II) the speed-down drying period (1–4 h for 80°C-HAD and VFD and 2–4 h for 60°C-HAD); and (III) the low-speed drying stage (4–8 h for 60°C-HAD, 80°C-HAD, and VFD). In the drying period I, the solid biomass medium presented higher WC and stronger heat absorption ability as compared to periods II and III, which caused the rapid water removal (7). With the extension of drying, the WC and heat absorption capacity of the medium were decreased. Meanwhile, the change in the internal structure of the solid medium also inhibited the water distribution (18), which was responsible for the DR decrease.

### Comparison of water state variations of solid media during different drying processes

**Figure 2:**
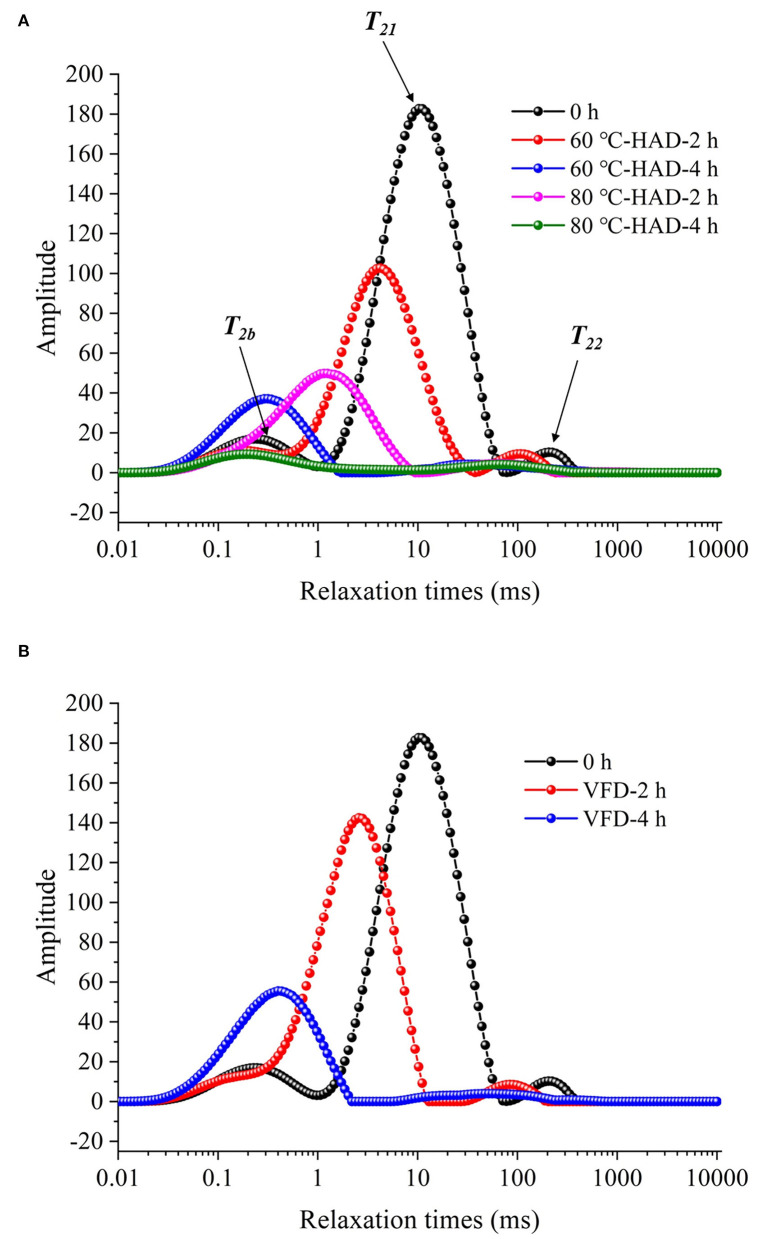
Development curves in relaxation time of solid medium with different drying strategies. **(A)** Hot air drying (HAD). 60°C-HAD-2 h: hot air drying at 60°C for 2 h; 60°C-HAD-4 h: hot air drying at 60°C for 4 h; 80°C-HAD-2 h: hot air drying at 80°C for 2 h; and 80°C-HAD-4 h: hot air drying at 80°C for 4 h. **(B)** Vacuum freeze-drying (VFD). VFD-2 h: vacuum freeze-drying for 2 h; VFD-4 h: vacuum freeze-drying for 4 h.

Low-field nuclear magnetic resonance can be used to evaluate water states in the substrate, which are significantly related to the microstructure of the solid medium (21). In this study, water dynamics and relaxation times of *T*_2*i*_ fractions were investigated to elucidate the microstructure changes of solid medium treated by different drying strategies. Figure 2 shows that three kinds of proton fractions are found in the solid medium after fermentation (curve at 0 h). The different fractions represent three different states of water, namely, (1) bound water, *T*_2*b*_ associated with hydroxyl and/or carboxyl hydrophilic groups (22); (2) capillary water, *T*_21_ attributed to the water molecules in pores of solid media (23); and (3) lumen water, *T*_22_ ascribed to water molecules in the cavities of the three-dimensional network in solid media (11). *M*_2*b*_, *M*_21_, and *M*_22_ were the corresponding water proportion, respectively. Table 1 shows that the *T*_2*b*_, *T*_21_, and *T*_22_ relaxation times and *M*_2*b*_, *M*_21_, and *M*_22_ water proportions are decreased during 60°C-HAD for 2 h, which indicates the reduction of water fluidity and a decrease of WC. *T*_21_ capillary water and *T*_22_ lumen water were the relatively free water in solid biomass medium, which can be removed first in the drying process. However, *T*_2*b*_ bound water was associated with hydroxyl and/or carboxyl hydrophilic groups, which was relatively difficult to remove. It should be noted that *T*_21_ and *T*_22_ peaks were merged into 1 weak peak at 60°C-HAD-4 h, 80°C-HAD-2 h, 80°C-HAD-4 h, and VFD-4 h conditions. *T*_2*b*_ and *T*_21_ peaks were merged into 1 weak peak at VFD-2 h. It was speculated that drying treatments can break the barriers between different water fractions, thereby leading to the merging of *T*_21_ and *T*_22_ and *T*_2*b*_ and *T*_21_ peaks. Tang et al. (24) also reported that water fractions can only be distinguished where microstructural barriers exist because they can hinder the inter-compartment water diffusion, which was consistent with the present results.

**Table 1:** Relaxation times (*T*_2*i*_) and water proportion (*M*_2*i*_) of the solid biomass medium during drying processes.

**[-67,13]6092.8ptDrying condition*T_2*i*_ and *M_2*i*_****	***T_2*b*_*(ms)**	***T_21_*(ms)**	***T_22_*(ms)**	***M_2*b*_*** ** (Peak area/a.u.)**	***M_21_*** ** (Peak area/a.u.)**	***M_22_*** ** (Peak area/a.u.)**	**∑*M_2*i*_*** ** (Peak area/a.u.)**
0 h	0.25	11.10	240.94	408	4630	125	5163
60°C-HAD-2 h	0.16	4.20	107.19	250	2701	65	3016
60°C-HAD-4 h	0.29	34.49 (T_21_T_22_M)		1023	140 (M_21_M_22_M)		1163
80°C-HAD-2 h	1.15	65.93 (T_21_T_22_M)		1617	107 (M_21_M_22_M)		1724
80°C-HAD-4 h	0.21	65.93 (T_21_T_22_M)		313	115 (M_21_M_22_M)		428
VFD-2 h	2.58 (T_2b_T_21_M)		84.07	3842 (M_2b_M_21_M)		116	3958
VFD-4 h	0.40	51.71 (T_21_T_22_M)		1601	132 (M_21_M_22_M)		1733

T_21_T_22_M: T_21_ and T_22_ merged; T_2b_T_21_M: T_2b_ and T_21_ merged; M_21_M_22_M: M_21_ and M_22_ merged; M_2b_M_21_M: M_2b_ and M_21_ merged; ∑M_2i_ is the sum of M_2b_, M_21_, and M_22_.

The ∑*M*_2*i*_ of the solid medium at 80°C-HAD-2 h decreased by 42.8% than that at 60°C-HAD-2 h, while the ∑*M*_2*i*_ at 80°C-HAD-4 h decreased by 63.2% than that at 60°C-HAD-4 h. These results indicated that the higher drying temperature during HAD could accelerate the water release from the medium. The proportion of *M*_2*b*_ to ∑*M*_2*i*_ at VFD-4 h was 92.4%, which was higher than that of 60°C-HAD and 80°C-HAD at the same drying time. VFD is a process in which water is crystallized at a low temperature and thereafter sublimated from the solid state directly into the vapor phase (25). The capillary water and lumen water were frozen as ice crystals and sublimated, but the bound water was retained with VFD. In summary, both HAD and VFD decreased the water fraction amplitudes and altered the water fraction distributions within a solid biomass medium. These different water states within the media should influence the sporulation process of *B. subtilis*.

### Expression of sporulation-regulatory genes during different drying processes

**Figure 3:**
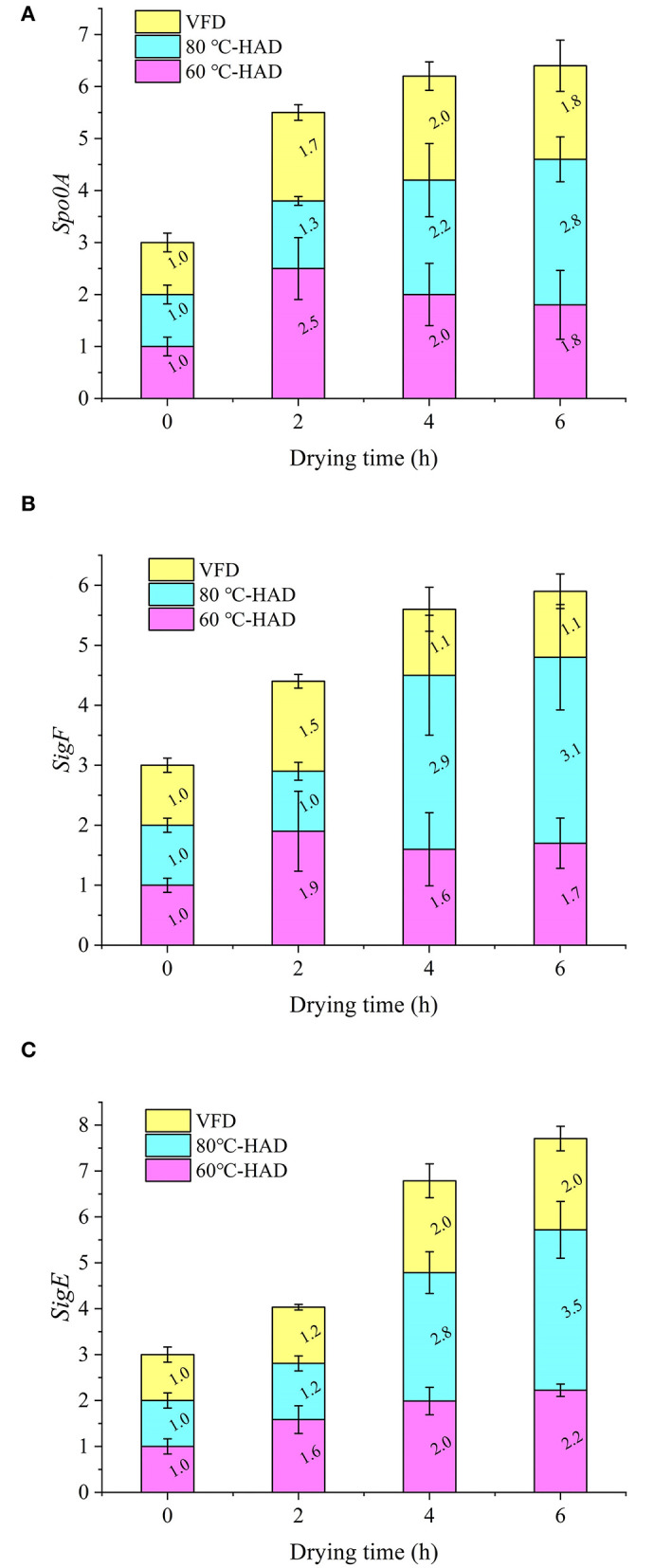
Expressions of *spo0A* **(A)**, *sigF* **(B)**, and *sigE* **(C)** genes during different drying processes.

The expression of *spo0A, sigF*, and *sigE* genes associated with sporulation during the drying processes was systematically investigated. Figure 3A shows that the expression of the *spo0A* gene exhibited an increasing tendency with the extension of drying time. Since the *spo0A* gene is the major regulator for the initiation of sporulation (26, 27), the increasing expression of *spo0A* illustrated the enhanced sporulation process with the HAD and VFD drying strategies. After drying for 6 h, the expression of the *spo0A* gene in 80°C-HAD reached 2.8, which was higher than that of 60°C-HAD (1.8) and VFD (1.8). It suggested that the 80°C-HAD drying strategy can facilitate the sporulation process more significantly.

As seen in Figures 3B,C, the expression of *sigF* and *sigE* genes in 80°C-HAD is increased by 206 and 249% from 0 to 6 h drying time, respectively. The expression of *sigF* and *sigE* genes in 60°C-HAD was increased by 70 and 120% from 0 to 6 h drying time, respectively. The expression of *sigF* and *sigE* genes in VFD increased by 10 and 100% from 0 to 6 h drying time, respectively. These results showed that the *sigF* and *sigE* genes exhibited increasing tendencies with the extension of drying time, which were similar to the *spo0A* gene expression variation. The expression levels of *sigF* and *sigE* gene at 80°C-HAD after 6 h drying were 1.76 and 1.57 times of those at 60°C-HAD. Fimlaid et al. (28) reported that the *sigF* and *sigE* genes are expressed in a *spo0A*-dependent manner. The higher the drying temperature, the more intense the sporulation process. Activation of the master transcriptional gene *spo0A* leads to the sequential activation of *sigF* and *sigE* genes, which can activate compartment-specific transcriptional programs that drive sporulation through its morphological stages (16). Both HAD and VFD induced sporulation. The expressions of *spo0A, sigF*, and *sigE* genes during drying followed the order 80°C-HAD > 60°C-HAD > VFD. These results indicated that high temperature in HAD facilitated sporulation-regulatory gene expression, which was more likely to promote sporulation of *B. subtilis*.

### Viable cell and spore number changes during different drying processes

**Figure 4:**
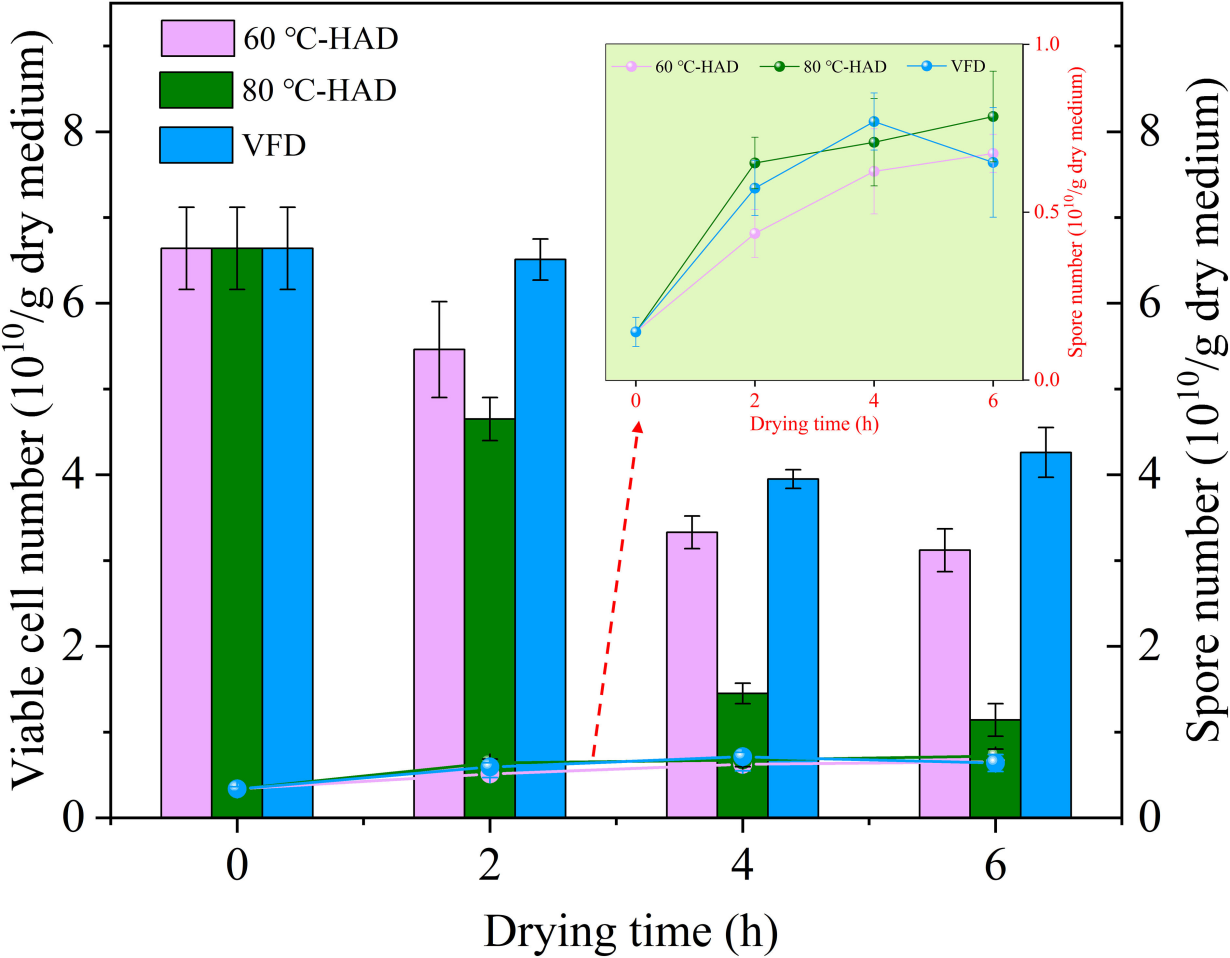
Comparison of viable cell and spore numbers during different drying processes.

The number of viable cells and spores can be used to evaluate the quality of microbial ecological agents, food additives, etc. (13, 29, 30). Figure 4 shows the variation of viable cell and spore numbers in media with different drying strategies. The viable cell number exhibited decreasing tendencies, while the spore number showed increasing trends with the extension of drying time in all media. It should be noted that the viable cell number decreased fast while the spore number increased rapidly from 0 to 4 h in 80°C-HAD, 60°C-HAD, and VFD processes. The possible reason for this phenomenon was that WC decreased fast during this drying period. Influenced by the rapid removal of water, the viable cell formed spores to resist harsh environments.

With the extension of drying time, the viable cell number of 80°C-HAD was always lower than that of 60°C-HAD, while the spore number of 80°C-HAD was always higher than that of 60°C-HAD. Especially, the viable cell numbers of 80°C-HAD at 4 and 6 h drying time were 1.45 × 10^10^ and 1.14 × 10^10^/g dry medium, respectively, which were lower than that of 60°C-HAD at 4 h (3.33 × 10^10^/g dry medium) and 6 h (3.12 × 10^10^/g dry medium). Moreover, the 80°C-HAD strategy caused a more rapid water decrease in the solid medium than 60°C-HAD, resulting in a more hydropenic environment. This severe condition induced more viable cell death or transformation to spores under 80°C-HAD. Previous studies reported that air drying caused physical alterations (e.g., shrinkage), which decreased the porosity of the microscopic structure of substrate materials (31–33). Similarly, HAD in the present study also led to the shrinkable structure of solid biomass medium with the extension of drying. The inferior porosity of the solid biomass medium was harmful to the growth of vegetative cells, which enhanced sporulation. The spore number of 80°C-HAD was 0.72 × 10^10^/g dry medium after 6 h of drying, which was 9.1 and 12.5% larger than that of 60°C-HAD and VFD, respectively. The spore number of 80°C-HAD, 60°C-HAD, and VFD was increased by 115, 95, and 90%, respectively, during 0–6 h drying processes. Therefore, it can be concluded that the drying processes in the present study promoted sporulation. It should also be noted that the viable cell number of VFD showed a slight increase while the spore number decreased slightly during drying for 4–6 h. This phenomenon was contrary to the general trend of viable cell and spore numbers but can be explained by the specific sublimation drying process of VFD. By drying in the frozen state, the free water fractions within the solid medium were presented as ice crystals. Then, these water fractions were sublimated continuously with the extension of drying time (25). As a result, the pores and cavities were vacated in the final period of drying, providing a porous medium and effective contact space for spore germination during 4–6 h drying time. Overall, VFD can maintain the porous structure of a solid medium, but HAD, especially 80°C-HAD, presented the vigorous sporulation process, which improved the titer of microbial ecological agents with *B. subtilis*.

**Figure 5:**
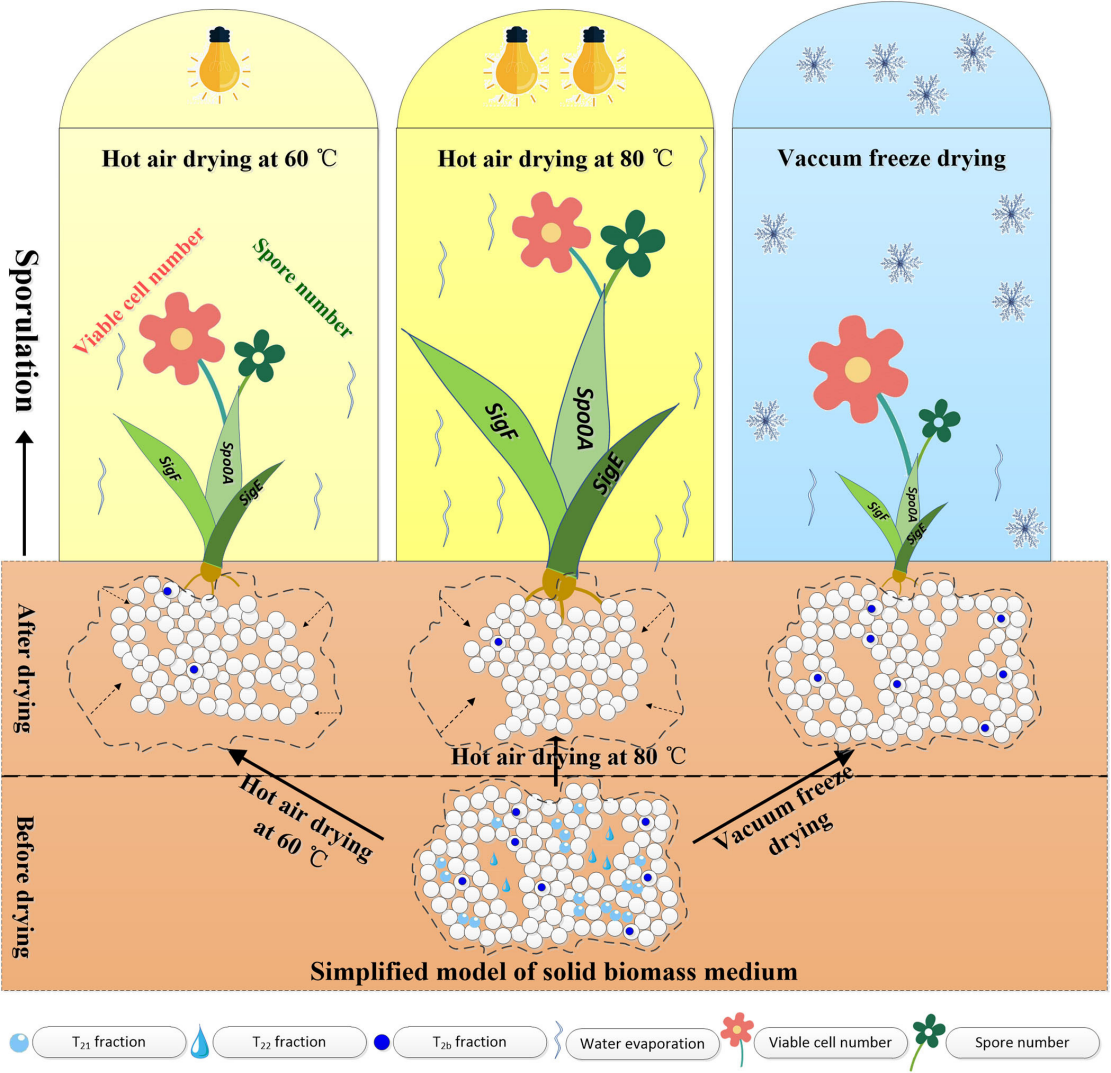
Proposed model for water state variations, microstructural changes of solid media, and *B. subtilis* sporulation during different drying processes.

Based on the above experimental results, a comprehensive model was proposed to elucidate the water state variations, microstructural changes of solid media, and *B. subtilis* sporulation during different drying processes. As seen in Figure 5, HAD leads to the shrinkage of pores and cavities in solid media, but VFD retains bound water and maintains the porous structure. Both HAD and VFD induced sporulation. The expression levels of *spo0A, sigF*, and *sigE* genes with 80°C-HAD were the highest as compared to 60°C-HAD and VFD, resulting in the largest spore number in medium with 80°C-HAD. The proposed model clearly elucidated the water states and microstructure changes within solid biomass media, which provided a better understanding of the effects of drying strategies on the sporulation of *B. subtilis*.

## Conclusion

Effects of different drying strategies on the sporulation of *B. subtilis* were revealed. Both HAD and VFD induced rapid water removal from solid biomass medium and facilitated sporulation. The expression level of sporulation-regulatory genes followed the order 80°C-HAD > 60°C-HAD > VFD. Accordingly, the spore number with 80°C-HAD was increased by 9.1 and 12.5% than that of 60°C-HAD and VFD, respectively. Therefore, 80°C-HAD is an effective drying strategy for promoting sporulation, which improves the titer of *B. subtilis* microbial ecological agents effectively.

## Data availability statement

The original contributions presented in the study are included in the article/supplementary material, further inquiries can be directed to the corresponding author/s.

## Author contributions

Conceptualization: Z-MZ. Formal analysis: CL, KZ, and Z-MZ. Funding acquisition: Z-MZ and LM. Investigation: KZ, CL, and Z-MZ. Project administration: Z-MZ and JZ. Writing—original draft: CL and KZ. Writing—review and editing. CL, KZ, LM, JZ, and Z-MZ. All authors contributed to the article and approved the submitted version.

## Funding

This work was supported by the National Natural Science Foundation of China (Grant Numbers 21706136 and 21766025) and the Natural Science Foundation of Inner Mongolia (Grant Number 2019MS02026).

## Conflict of interest

The authors declare that the research was conducted in the absence of any commercial or financial relationships that could be construed as a potential conflict of interest.

## Publisher's note

All claims expressed in this article are solely those of the authors and do not necessarily represent those of their affiliated organizations, or those of the publisher, the editors and the reviewers. Any product that may be evaluated in this article, or claim that may be made by its manufacturer, is not guaranteed or endorsed by the publisher.

## References

[1] Guan AM Qi WX Peng Q Zhou JM Bai YH Qu JH. Environmental heterogeneity determines the response patterns of microbially mediated N-reduction processes to sulfamethoxazole in river sediments. J Hazard Mater. (2021) 11:126730. 10.1016/j.jhazmat.2021.126730 34388921

[2] Barrios RE Khuntia HK Bartelt-Hunt SL Gilley JE Schmidt AM Snow DD Li X. Fate and transport of antibiotics and antibiotic resistance genes in runoff and soil as affected by the timing of swine manure slurry application. Sci Total Environ. (2020) 712:136505. 10.1016/j.scitotenv.2020.136505 31931227

[3] Pan X Cai YL Kong LL Xiao CP Zhu QD Song ZG. Probiotic effects of *Bacillus licheniformis* DSM5749 on growth performance and intestinal microecological balance of laying hens. Front Nutr. (2022) 9:868093. 10.3389/fnut.2022.868093 35571886 PMC9093703

[4] Kaspar F Neubauer P Gimpel M. Bioactive secondary metabolites from *Bacillus subtilis*: a comprehensive review. J Nat Prod. (2019) 82:7. 10.1021/acs.jnatprod.9b00110 31287310

[5] Gao DY Sun XB Liu MQ Liu YN Zhang HE Shi XL. Characterization of thermostable and chimeric enzymes via isopeptide bond-mediated molecular cyclization. J Agr Food Chem. (2019) 67:6837–46. 10.1021/acs.jafc.9b01459 31180217

[6] Chen H Chen SW Li CN Shu GW. Response surface optimization of lyoprotectant for *lactobacillus bulgaricus* during vacuum freeze-drying. Prep Biochem Biotech. (2015) 45:463–75. 10.1080/10826068.2014.923451 24840953

[7] Ambros S Mayer R Schumann B Kulozik U. Microwave-freeze drying of lactic acid bacteria: Influence of process parameters on drying behavior and viability. Innov Food Sci Emerg. (2018) 48:90–8. 10.1016/j.ifset.2018.05.02022266474

[8] Byun SY Kang JS Chang YS. Analysis of primary drying of poly-γ-glutamic acid during vacuum freeze drying. J Mech Sci Technol. (2020) 34:4323–32. 10.1007/s12206-020-0922-9

[9] Kirtil E Oztop MH. ^1^H nuclear magnetic resonance relaxometry and magnetic resonance imaging and applications in food science and processing. Food Eng Rev. (2015) 8:1–22. 10.1007/s12393-015-9118-y

[10] Kirtil E Cikrikci S Mccarthy MJ Oztop MH. Recent advances in time domain NMR & MRI sensors and their food applications. Curr Opin Food Sci. (2017) 17:9–15. 10.1016/j.cofs.2017.07.005

[11] Li T Tu C Rui X Gao Y Li W Wang K. Study of water dynamics in the soaking, steaming, and solid-state fermentation of glutinous rice by LF-NMR: a novel monitoring approach. J Agr Food Chem. (2015) 63:3261–70. 10.1021/acs.jafc.5b00769 25775016

[12] Cheng SS Li RR Yang HM Wang SQ Tan MQ. Water status and distribution in shiitake mushroom and the effects of drying on water dynamics assessed by LF-NMR and MRI. Dry Technol. (2020) 38:1001–10. 10.1080/07373937.2019.1625364

[13] Veen B Xie H Esveld E Abee T Mastwijk H Groot MN. Inactivation of chemical and heat-resistant spores of *Bacillus* and *Geobacillus* by nitrogen cold atmospheric plasma evokes distinct changes in morphology and integrity of spores. Food Microbiol. (2015) 45:26–33. 10.1016/j.fm.2014.03.018 25481059

[14] Setlow P Johnson EA. Spores and their significance. Food Microbiol Fundam Front. (2019) 23–63. 10.1128/9781555819972.ch2

[15] Zhao ZM Xi JT Xu JF Ma LT Zhao J. Enhancement of *Bacillus subtilis* growth and sporulation by two-stage solid-state fermentation strategy. Processes. (2019) 7:644. 10.3390/pr7100644

[16] Tan IS Ramamurthi KS. Spore formation in *Bacillus subtilis*. Env Microbiol Rep. (2014) 6:212–25. 10.1111/1758-2229.12130 24983526 PMC4078662

[17] Patel SM Doen T Pikal MJ. Determination of end point of primary drying in freeze-drying process control. AAPS PharmSciTech. (2010) 11:73–84. 10.1208/s12249-009-9362-7 20058107 PMC2850457

[18] Sun Y Zhang M Mujumdar AS Yu D. Pulse-spouted microwave freeze drying of raspberry: control of moisture using ANN model aided by LF-NMR. J Food Eng. (2021) 292:110354. 10.1016/j.jfoodeng.2020.110354

[19] Farrag M Abri S Leipzig ND. pH-dependent RNA isolation from cells encapsulated in chitosan-based biomaterials. Int J Biol Macromol. (2020) 146:422–30. 10.1016/j.ijbiomac.2019.12.263 31904458 PMC7029618

[20] Hong Y Zhou Z Yu LZ Jiang KY Xia JM Mi YL Zhang CQ Li J. *Lactobacillus salivarius* and *lactobacillus agilis* feeding regulates intestinal stem cells activity by modulating crypt niche in hens. Appl Microbiol Biot. (2021) 105:8823–35. 10.1007/s00253-021-11606-2 34708278

[21] Liu ZH Chen HZ. Biomass-water interaction and its correlations with enzymatic hydrolysis of steam-exploded corn stover. ACS Sustain Chem Eng. (2016) 4:1274–85. 10.1021/acssuschemeng.5b01303

[22] Li T Rui X Li W Chen XH Jiang M Dong MS. Water distribution in tofu and application of *T[[sb]]2[[/s]]* relaxation measurements in determination of Tofu's water-holding capacity. J Agr Food Chem. (2014) 62:8594–601. 10.1021/jf503427m 25094026

[23] Felby C Thygesen LG Kristensen JB Jrgensen H Elder T. Cellulose-water interactions during enzymatic hydrolysis as studied by time domain NMR. Cellulose. (2008) 15:703–10. 10.1007/s10570-008-9222-8

[24] Tang HR Godward J Hills B. The distribution of water in native starch granules—a multinuclear NMR study. Carbohyd Polym. (2000) 43:375–87. 10.1016/S0144-8617(00)00183-1

[25] Liu Y Zhao Y Feng X. Exergy analysis for a freeze-drying process. Appl Thermal Eng. (2008) 28:675–90. 10.1016/j.applthermaleng.2007.06.004

[26] Molle V Fujita M Jensen ST Eichenberger P Losick R. The spo0A regulon of *bacillus subtilis*. Mol Microbiol. (2004) 50:1683–701. 10.1046/j.1365-2958.2003.03818.x 14651647

[27] Fujita M Gonzalez-Pastor JE Losick R. High- and low-threshold genes in the Spo0A regulon of *Bacillus subtilis*. J Bacteriol. (2005) 187:1357–68. 10.1128/JB.187.4.1357-1368.2005 15687200 PMC545642

[28] Fimlaid KA Shen A. Diverse mechanisms regulate sporulation sigma factor activity in the firmicutes. Curr Opin Microbiol. (2015) 24:88–95. 10.1016/j.mib.2015.01.006 25646759 PMC4380625

[29] Velmourougane K Prasanna R Saxena AK. Agriculturally important microbial biofilms: Present status and future prospects. J Basic Microbiol. (2017) 57:548–73. 10.1002/jobm.201700046 28407275

[30] Mohsin MZ Omer R Huang J Mohsin A Zhuang Y. Advances in engineered *Bacillus subtilis* biofilms and spores, and their applications in bioremediation, biocatalysis, and biomaterials. Syn Syst Biotechno. (2021) 6:180–91. 10.1016/j.synbio.2021.07.002 34401544 PMC8332661

[31] Ratti C. Hot air and freeze-drying of high-value foods: a review. J Food Eng. (2001) 49:311–9. 10.1016/S0260-8774(00)00228-4

[32] Witrowa-Rajchert D Lewicki PP. Rehydration properties of dried plant tissues. Int J Food Sci Technol. (2006) 41:1040–6. 10.1111/j.1365-2621.2006.01164.x

[33] Ciurzyńska A Lenart A. Freeze-drying-application in food processing and biotechnology–a review. Pol J Food Nutr Sci. (2011) 61:3. 10.2478/v10222-011-0017-5

